# Analysis of indications for selectively missing results in comparative registry-based studies in medicine: a meta-research study

**DOI:** 10.1186/s41073-025-00159-x

**Published:** 2025-03-05

**Authors:** Paula Starke, Zhentian Zhang, Hannah Papmeier, Dawid Pieper, Tim Mathes

**Affiliations:** 1 Department of Medical Statistics, University Medical Center Göttingen, Göttingen, Germany; 2 Faculty of Health Sciences Brandenburg, Brandenburg Medical School (Theodor Fontane), Institute for Health Services and Health System Research, Rüdersdorf, Germany; 3 Center for Health Services Research, Brandenburg Medical School (Theodor Fontane), Rüdersdorf, Germany; 4 Evidence Based Practice in Brandenburg: A JBI Affiliated Group, University of Adelaide, Adelaide, Australia

**Keywords:** Reporting bias, Registration, Selective reporting, Publication bias, P-hacking, Registries, Effectiveness, Cohort study

## Abstract

**Background:**

We assess if there are indications that results of registry-based studies comparing the effectiveness of interventions might be selectively missing depending on the statistical significance (*p* < 0.05).

**Methods:**

**Eligibility criteria** Sample of cohort type studies that used data from a patient registry, compared two study arms for assessing a medical intervention, and reported an effect for a binary outcome. **Information sources** We searched PubMed to identify registries in seven different medical specialties in 2022/23. Subsequently, we included all studies that satisfied the eligibility criteria for each of the identified registries and collected *p*-values from these studies. **Synthesis of results** We plotted the cumulative distribution of *p*-values and a histogram of absolute z-scores for visual inspection of selectively missing results because of p-hacking, selective reporting, or publication bias. In addition, we tested for publication bias by applying a caliper test.

**Results:**

**Included studies** Sample of 150 registry-based cohort type studies. **Synthesis of results** The cumulative distribution of *p*-values displays an abrupt, heavy increase just below the significance threshold of 0.05 while the distribution above the threshold shows a slow, gradual increase. The *p*-value of the caliper test with a 10% caliper was 0.011 (k = 2, *N* = 13).

**Conclusions:**

We found that the results of registry-based studies might be selectively missing. Results from registry-based studies comparing medical interventions should be interpreted very cautiously, as positive findings could be a result from p-hacking, publication bias, or selective reporting. Prospective registration of such studies is necessary and should be made mandatory both in regulatory contexts and for publication in journals. Further research is needed to determine the main reasons for selectively missing results to support the development and implementation of more specific methods for preventing selectively missing results.

**Supplementary Information:**

The online version contains supplementary material available at 10.1186/s41073-025-00159-x.

## Background

The amount of registry-based medical research has increased over the last decades [1]. Meanwhile, registries are often used to generate real-world evidence by comparing the effectiveness of interventions including their application in health technology assessments [2, 3].

Analyses of registry data are observational. Observational studies are usually not prospectively registered and prospective specification of outcomes and statistical analyses rarely occur [4]. This makes them suspicious of selectively missing results because of p-hacking, selective reporting, or publication bias [5–7]. The risk for selectively missing results is especially high in registry-based studies because the analysis is usually planned after data collection, which increases the risk of various biases, including the selection of results from various analysis strategies (i.e. p-hacking and selective reporting) [8].

Selectively missing negative and null findings can result in an overestimation of estimates for the intervention effect and thus in making wrong regulatory or treatment decisions. Furthermore, this contributes to wasted research resources because findings are not available to inform future research.

We aimed to assess if there are indications that results of registry-based studies comparing the effectiveness of medical interventions might be selectively missing depending on the statistical significance (*p* < 0.05).

## Methods

The protocol of this study was registered in the open science framework: https://osf.io/m6s2b/. Data and the R script used for the analyses can be found on Gitlab (ID: 41,680, https://gitlab.gwdg.de/starke10/PubBias/). We followed the Preferred Reporting Items for Systematic reviews and Meta-Analyses (PRISMA) as far as applicable [9].

### Eligibility criteria

All cohort type studies that used data from a patient registry, compared two study arms for assessing a medical intervention and reported an effect for a binary outcome were included. We focused on studies assessing intervention effects because of its increasing importance for medical decision-making including health-technology assessment. Furthermore, we anticipated that considering also registry-based studies on other medical questions (e.g. risk factors) would have resulted in very large heterogeneity, as the analyses methods and publication process are different [2, 3].

To ensure a consistent study selection we defined a registry as “an electronic database containing uniform information about individual persons, collected in a systematic way, in order to serve a predetermined purpose” [10]. We only considered patient registries. Health system registries, mortality registries, registries of residents and other administrative registries were excluded.

### Data source and study selection

Our aim was to get an overview of selective reporting across different medical disciplines. In the sense of stratified sampling, we applied an iterative two-step approach for identifying relevant registry-based studies to achieve this. In the first step, we systematically searched all PubMed databases for recently conducted registry-based cohort studies and compiled a list of the identified registries. We continued the search starting from the most recently published study and subsequently proceeded to the preceding publication until we identified a registry for each of the following disciplines: accident and emergency medicine, cardiology, endocrinology/diabetes/ metabolism, general surgery, infectious diseases, oncology, orthopedics, pediatrics and psychiatry.

The first eligible registry for each of the medical specialties was included. For identifying registry-based studies, we developed an electronic search strategy that combines terms for registry data with a validated sensitive search filter for non-randomized comparative study designs [see Additional file 1] [11]. Additionally, the search strategy included the medical subject headings (MeSH) “therapeutics” or “surgical procedures, operative" to further narrow the search to studies that assess a medical intervention. We identified eligible patient registries by screening studies published in the 3 months prior to each search date (14/01/2022–14/04/2022 and 09/08/2022–09/11/2022).

In the second step, PubMed was searched once for each included registry (between 05/2022 and 03/2023) by the official name of the registry as well as any acronyms and abbreviations of the registry name to identify cohort type studies comparing an intervention. If a search using the registry name retrieved more than 200 hits, we added the more specific search filter and MeSH terms that were used in step one [11]. For each of the registries, we required that at least ten studies were available for inclusion in the analysis.

The identification of the registries and selection of studies was performed using the online tool rayyan.ai [12] by two reviewers independently.

### Data collection

Studies that are based on registry data often compare more than two interventions and assess several outcomes. We only extracted the results of the primary comparison and outcome. If the primary clinical question was not clear, we extracted data for the comparison that appears first in the results section of the abstract or the full publication.

Descriptive data for each study included population characteristics, description of intervention and control, endpoint definition, the number of patients, events per group, and effect estimates with precision or *p*-values. If no exact *p*-value was reported, we calculated the *p*-values from the confidence intervals. In addition, we checked if the studies were registered in a study registry, or if a published study protocol was referenced.

Data were extracted by one statistician and checked by a second statistician.

### Analysis of selectively missing results

The characteristics of the included studies were described using percentages.

We applied various methods to assess if the results were suggestive of selectively missing results. For visual inspection, we plotted the cumulative distribution of *p*-values (to avoid the sensitivity to bin width of p-curves plotted as histograms or kernel density estimates) and a histogram of the absolute two-sided z-scores calculated from the *p*-values.

In the case of no selectively missing results, and if in truth no intervention effect exists in a sample of independent studies, the cumulative density should be a line of slope 1. When a true intervention effect is present, we would instead expect a smooth, gradually increasing concave function as the density function will be right skewed with an increased density for lower *p*-values (see [13] for an example). Some irregularities can be expected because of the different sizes of the intervention effect, which would only smooth out in a very large sample.

In our sample of different clinical questions, we would expect a mixture of true intervention effects, different effect sizes, and null effects. Thus, the curve we observe should be in between the described two curves. The density plot should look similar to curves empirically observed for pre-registered randomized controlled trials [14]. Compared to a curve for RCTs the right skew of the density curve for registry-based studies can be expected to be sharper as registry-based studies are often large and not powered to a minimal relevant effect size. However, there should be no conspicuous irregularities around the common thresholds for statistical significance in the curves when the results are completely free from active selection of results. In contrast, in the case of selectively missing results depending on the statistical significance level due to selective reporting, publication bias or p-hacking, we would expect an irregularity in the curve shape particularly near the widely accepted *p*-value of *p* < 0.05. More specifically, we would expect a sharp step, or pulse.

We prepared plots of the cumulative frequency of *p*-values and histograms of absolute z-scores both across all studies and for each registry separately.

In addition, we tested for publication bias using a caliper test [15]. We decided to use this test because it is appropriate and has an intuitive interpretation also for heterogeneous effect sizes [16]. Other common methods used for assessing publication bias in meta-analysis, like tests related to the funnel-plot [17–19], the p-curve [20] and the z-curve [21] are not adequate or more difficult to interpret for largely heterogeneous effect sizes that come from a mixture of different clinical questions from various medical specialties like in our case.

The null hypothesis of the caliper test states that without publication bias, a z score just above and just below the significance threshold should be about equally likely. We used a 10% caliper. The width of the caliper indicates that z-values that are 10% smaller or larger than the critical value (1.96 for two-tailed tests) are included in the analysis. As a sensitivity analysis, we computed the caliper test in the subset of studies for which exact confidence intervals for OR, RR or HR measures were available and thus exact z-statistics could be computed.

### Patient and public involvement

We did not involve patients or members of the public when we designed the study or interpreted the results.

## Results

### Literature search

**Fig. 1:**
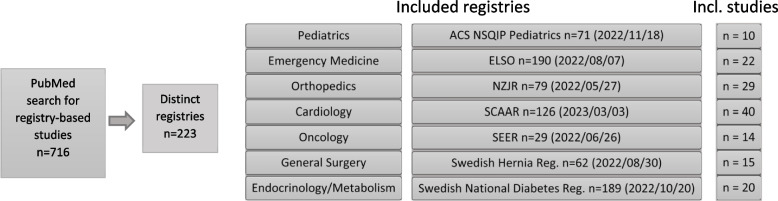
Flow-chart for identification of registries and studies

The flow-chart in Fig. 1 illustrates the literature search process for the identification of eligible registries and the corresponding number of registry-based studies. For psychiatry and infectious diseases, we could not identify any registry that included a least 10 studies comparing a medical intervention in the first or second search wave. One registry from each of the other medical specialties was included. In total 150 studies were included.

Table 1 shows the characteristics of the included studies. The complete extraction table is provided as an additional file [see Additional file 2]. Most (59%) of the studies include more than 5000 patients. The majority of studies compared surgical procedures (40%) or medical devices (38%). Comparisons of drugs (18%) or other types of interventions (4%) were less frequent. As many studies were on cardiology (27%) and orthopaedic registries (19%), the most frequently analysed outcome measures were the revision rate (25%), the occurrence of cardiovascular events (20%) and mortality (25%). More than half of the studies used some kind of Cox regression (53%). Logistic regression (12%) was the most frequently applied analysis for studies not using time-to-event analyses. For 16 studies (11%), the method of analysis was not explicitly stated in the publication. Only 4 out of 150 studies were pre-registered (3%).

**Table 1:** Characteristics of included registry-based comparative studies

	***N* = 150**
**Population size**	
≤ *500*	10
* 501—1 500*	22
* 1 501—5 000*	29
* 5 001—50 000*	59
> *50 000*	24
* Not reported*	6
**Intervention**	
* Surgical procedure*	60
* Medical device*	57
* Drug*	27
* Other*	6
**Method of analysis**	
* Cox regression*	79
* Logistic regression*	18
* Chi-square test*	14
* Log-rank test*	8
* Instrumental variable analysis*	2
* T-test*	2
* Other*	11
* Not reported*	16
**Outcome measures**	
* Revision rate*	37
* Cardiovascular events*	30
* Mortality*	37
* Composite adverse events*	18
* Other health outcomes*	15
* Other complications*	6
* Other*	7
**Type of effect estimate**	
* Hazard ratio*	76
* Odds ratio*	29
* Difference in means*	21
* Risk ratio*	15
* Other*	2
* Not reported*	7
**Pre-Registration or published protocol**	4

### Selectively missing results in registry-based studies

**Fig. 2:**
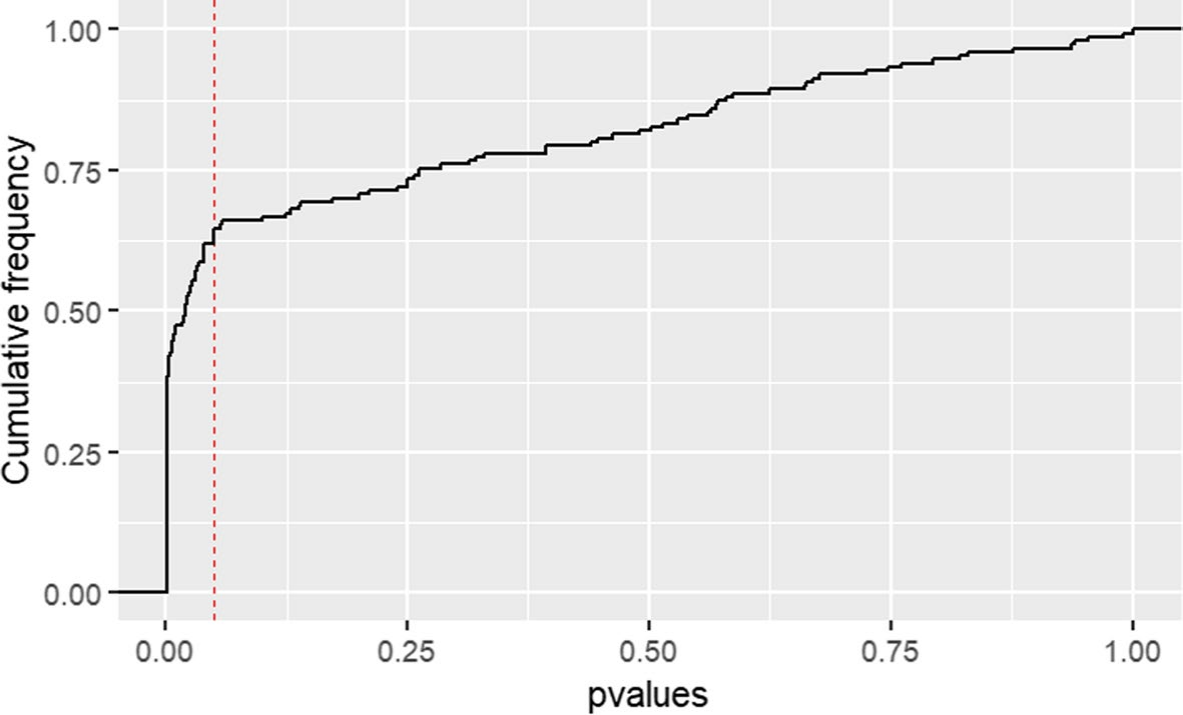
cumulative distribution of *p*-values; significance threshold of p = 0.05 is marked by the red line

The cumulative distribution of *p*-values is shown in Fig. 2. Thirty-six *p*-values were calculated from confidence intervals because no *p*-value was reported and one value because no exact *p*-value was reported. For one *p*-value no confidence interval was given and it was also reported only as ‘*p* < 0.05’. Therefore, we imputed it with the bootstrapped mean of all significant values.

We observe an abrupt heavy increase just at *p* < 0.05. There was no indication that this pattern of an abrupt increase of the number of *p*-values just below the 5%-threshold was markedly different for any of the registries [see Additional file 3].

**Fig. 3:**
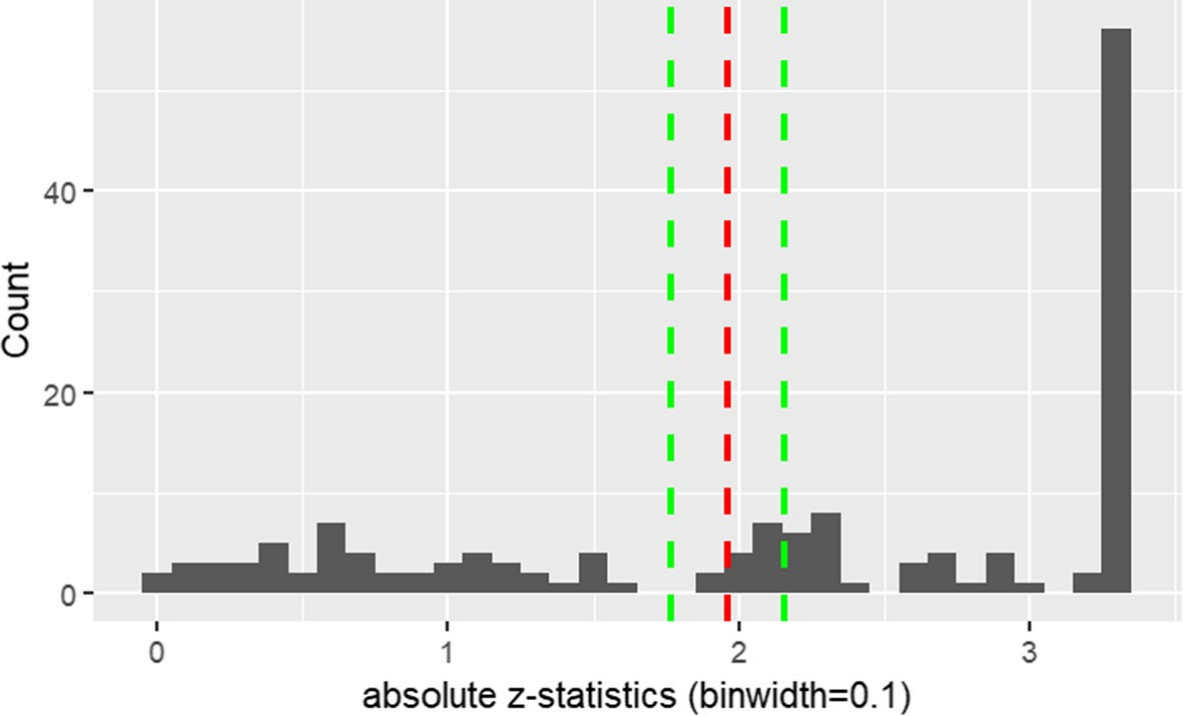
histogram of absolute z-scores; significance threshold of z = 1.96 in red; 10%-caliper in green; binwidth = 0.1

Figure 3 displays a histogram of the absolute z-scores. It shows that values just before the common thresholds for statistical significance are missing.

We observed significantly more z-scores just above 1.96 than just below. The *p*-value of the caliper test with a 10% caliper [1.76 < z- score ≤ 2.16] was 0.011 (k = 2, *N* = 13), confirming the visible violation of continuity in a narrow interval around the 1.96 threshold. The *p*-value of the caliper test in the reduced sample of only 109 studies, for which exact z-values could be calculated from confidence intervals, was 0.113 (k = 3, *N* = 11). It thus indicates the same pattern but with lower confidence. This could be expected because of reduced power due to the smaller sample size.

## Discussion

To our knowledge, this is the first study that assesses indications of selectively missing results in registry-based studies comparing a medical intervention. We focused on registry-based studies because of the increasing importance of registry-based medical research [1]. Furthermore, we anticipated that due to the open-ended data collection and often missing pre-specification of research questions in registry-based studies the risk of selectively missing results is higher than for other comparative observational studies like cohort studies.

We found that the results of registry-based studies might be selectively missing. The cumulative p-curve showed an abrupt heavy step below the threshold of *p* < 0.05. In addition, the *p*-value of the caliper test means that the probability of observing *p*-values equal to or more extreme than the ones observed is only 0.011.

Only very few of the included studies referred to a registry entry or a published protocol and thus it cannot be checked if the reported results are in agreement with the initially planned primary hypothesizes and statistical analysis plans. This registration rate is even less than that of observational studies in general [4].

The observed large number of small *p*-values may have several reasons. On the one hand, a large number of small *p*-values appears to be justified as usually some expectation on the intervention effect exists before a registry-based study is conducted, and consequently research questions are likely pre-selected in the way that comparisons with a higher chance of showing an (statistical significant) effect are investigated more often. Due to this pre-selection of questions, it can be expected that there tend to be more studies with true effects than studies with null effects and likewise a relative higher proportion of small *p*-values. However, this is also true for RCTs. While this phenomenon of pre-selecting questions based on chance of success potentially limits the scope of research, it is not a critical research practice. Furthermore, the large sample sizes of many registry-based studies might lead to very small *p*-values in those studies with a true effect. On the other hand, questionable research practices could be an explanation. It is particularly spurious that the irregularities can be observed exactly around the common thresholds for statistical significance. In addition, although there are some plausible explanations (see above) for more (very) small *p*-values compared to the curve for RCTs, the much heavier increase of the *p*-value plot curve seems a little suspicious.

We could not distinct if p-hacking, publication bias, or selective reporting is the main reason for systematically missing *p*-values. Most of the included studies used some kind of regression analysis. When using regression models, not only the outcome variable can be manipulated (e.g. different categorizations, handling missing data), but also other parameters like the covariates. Studies on p-hacking suggest that regression analyses are vulnerable to p-hacking. Particularly, selective inclusion and operationalization of covariates can potentially result in high false discovery rates [5]. Publication bias and selective reporting are prevalent in medical research [22, 23].

It appears likely that the selective missing *p*-values originate from a mixture of all of the potential sources with unknown weights. Further research is needed to explore the main reason for spurious findings, namely p-hacking, selective reporting or publication bias. This could support the development and implementation of more specific methods for preventing selectively missing results.

Currently, there are no effective measures to prevent the selective omission of results in registry-based studies comparing interventions. Prospective registration of studies used for assessing the comparative effectiveness of interventions would increase transparency and can help to reduce unreliable research findings [24]. Strategies for preventing p-hacking, and selective publication and reporting are necessary. For example, funders or holders of registries could require obligatory registration and publication of all studies that are based on their registry. If used for regulatory decisions, the pre-registration of a study protocol should be made mandatory. Journal editors should include this as a formal requirement. In addition, standards on how to design protocols for such registry-based studies should be refined. As there is a large number of strategies for manipulating *p*-values that can dramatically increase false discovery rates, very detailed study protocols and statistical analysis plans appear to be necessary for effectively preventing p-hacking. Study protocol templates for real-world evidence studies have already been developed [25].

Implementing measures to avoid selectively missing results of registry-based research is particularly important because publications of such studies will likely increase further due to the digitalization of healthcare systems and the concomitant generation of routinely collected data.

### Limitations

Our work has several limitations. Some medical specialities and registries were overrepresented in our sample. Specifically, we included an excess number of studies from the cardiology registry. In addition, we could not identify a registry for psychology and infectious disease. The results might be different for another sample. However, considering that the observed pattern of an abrupt increase of the number of *p*-values just below the 5%-threshold is visible across most different specialities/registries, the findings will probably hold for other registries.

We cannot completely rule out that the observed p-curve is a result of very high power. However, such a high average power seems unlikely in practice.

## Conclusion

We found indications for selectively missing results in our sample of comparative registry-based studies in medicine. The reasons maybe p-hacking, publication bias, or selective reporting. The indications for selectively missing results are particularly worrying because for almost no study a registry entry, or study protocol existed, which means that usually the credibility of the results cannot be checked.

The possibility that the results maybe too positive should be considered in the interpretation of comparative registry-based studies in medicine.

## Supplementary Information

Additional file 1. Search strings for selecting registry-based cohort studies comparing interventions.

Additional file 2. Data table containing all information that were extracted for included studies.

Additional file 3. Sub-Plots for single registries.

## Data Availability

The search strings and a table with extracted data for the included studies are included in the supplementary information files.

## Abbreviations

MeSH: Medical Subject Headings
PRISMA: Preferred Reporting Items for Systematic reviews and Meta-Analyses

## References

[1] Romanini E, Schettini I, Torre M, Venosa M, Tarantino A, Calvisi V, et al. The rise of registry-based research: a bibliometric analysis. Acta Orthop. 2021;92(5):628–32. 34139929 10.1080/17453674.2021.1937459 PMC8522812

[2] Mandeville KL, Valentic M, Ivankovic D, Pristas I, Long J, Patrick HE. QUALITY ASSURANCE OF REGISTRIES FOR HEALTH TECHNOLOGY ASSESSMENT. Int J Technol Assess Health Care. 2018;34(4):360–7. 30251946 10.1017/S0266462318000478

[3] Gliklich R, Dreyer NA, Leavy MB, Velentgas P, Khurana L. Standards in the Conduct of Registry Studies for Patient-Centered Outcomes Research: A Guidance Document for the Patient-Centered Outcomes Research Institute2012 2024 March 12 2024 March 12]:[58 p.]. Available from: https://www.pcori.org/assets/Standards-in-the-Conduct-of-Registry-Studies-for-Patient-Centered-Outcomes-Research.pdf.

[4] Boccia S, Rothman KJ, Panic N, Flacco ME, Rosso A, Pastorino R, et al. Registration practices for observational studies on ClinicalTrials.gov indicated low adherence. J Clin Epidemiol. 2016;70:176–82. 26386325 10.1016/j.jclinepi.2015.09.009

[5] Stefan AM, Schoenbrodt FD. Big little lies: a compendium and simulation of p-hacking strategies. Roy Soc Open Sci. 2023;10(2):9–13. 10.1098/rsos.220346 PMC9905987 36778954

[6] Thomas ET, Heneghan C. Catalogue of bias: selective outcome reporting bias. BMJ Evidence-Based Medicine. 2022;27(6):370–2. 10.1136/bmjebm-2021-111845 35177482

[7] DeVito NJ, Goldacre B. Catalogue of bias: publication bias. BMJ Evidence-Based Medicine. 2019;24(2):53–4. 10.1136/bmjebm-2018-111107 30523135

[8] Sterne JA, Hernán MA, Reeves BC, Savović J, Berkman ND, Viswanathan M, et al. ROBINS-I: a tool for assessing risk of bias in non-randomised studies of interventions. BMJ. 2016;355: i4919. 27733354 10.1136/bmj.i4919 PMC5062054

[9] Page MJ, McKenzie JE, Bossuyt PM, Boutron I, Hoffmann TC, Mulrow CD, et al. The PRISMA 2020 statement: an updated guideline for reporting systematic reviews. BMJ. 2021;372: n71. 33782057 10.1136/bmj.n71 PMC8005924

[10] Mathes T, Zhang Z, Pachanov A, Pieper D. Systematic reviews and meta-analyses that include registry-based studies: methodological challenges and areas for future research. J Clin Epidemiol. 2023;156:119–22. 36806731 10.1016/j.jclinepi.2023.02.014

[11] Waffenschmidt S, Navarro-Ruan T, Hobson N, Hausner E, Sauerland S, Haynes RB. Development and validation of study filters for identifying controlled non-randomized studies in PubMed and Ovid MEDLINE. Res Synth Methods. 2020;11(5):617–26. 32472632 10.1002/jrsm.1425

[12] Ouzzani M, Hammady H, Fedorowicz Z, Elmagarmid A. Rayyan—a web and mobile app for systematic reviews. Syst Rev. 2016;5(1):210. 27919275 10.1186/s13643-016-0384-4 PMC5139140

[13] Bishop DV, Thompson PA. Problems in using p-curve analysis and text-mining to detect rate of p-hacking and evidential value. PeerJ. 2016;4: e1715. 26925335 10.7717/peerj.1715 PMC4768688

[14] Belas N, Bengart P, Vogt B. P-hacking in Clinical Trials: A Meta-Analytical Approach. Working Paper Series [Internet]. 2017 2024 March 12; 19/2017. Available from: https://www.fww.ovgu.de/fww_media/femm/femm_2017/2017_19-p-8998.pdf.

[15] Gerber AS, Malhotra N. Publication Bias in Empirical Sociological Research: Do Arbitrary Significance Levels Distort Published Results? Sociological Methods & Research. 2008;37(1):3–30.

[16] Schneck A. Examining publication bias-a simulation-based evaluation of statistical tests on publication bias. PeerJ. 2017;5: e4115. 29204324 10.7717/peerj.4115 PMC5712469

[17] Duval S, Tweedie R. Trim and Fill: A Simple Funnel-Plot–Based Method of Testing and Adjusting for Publication Bias in Meta-Analysis. Biometrics. 2004;56(2):455–63. 10.1111/j.0006-341x.2000.00455.x 10877304

[18] Egger M, Smith GD, Schneider M, Minder C. Bias in meta-analysis detected by a simple, graphical test. BMJ. 1997;315(7109):629–34. 9310563 10.1136/bmj.315.7109.629 PMC2127453

[19] Begg CB, Mazumdar M. Operating characteristics of a rank correlation test for publication bias. Biometrics. 1994;50:1088–101. 7786990

[20] Simonsohn U, Nelson LD, Simmons JP. P-curve: a key to the file-drawer. J Exp Psychol Gen. 2014;143(2):534. 23855496 10.1037/a0033242

[21] Bartoš F, Schimmack U. Z-curve 2.0: Estimating replication rates and discovery rates. Meta-Psychology. 2022;6:10–11.

[22] Song F, Parekh-Bhurke S, Hooper L, Loke YK, Ryder JJ, Sutton AJ, et al. Extent of publication bias in different categories of research cohorts: a meta-analysis of empirical studies. BMC Med Res Methodol. 2009;9(1):79. 19941636 10.1186/1471-2288-9-79 PMC2789098

[23] Chan A-W, Hróbjartsson A, Haahr MT, Gøtzsche PC, Altman DG. Empirical Evidence for Selective Reporting of Outcomes in Randomized TrialsComparison of Protocols to Published Articles. JAMA. 2004;291(20):2457–65. 15161896 10.1001/jama.291.20.2457

[24] Naudet F, Patel CJ, DeVito NJ, Goff GL, Cristea IA, Braillon A, et al. Improving the transparency and reliability of observational studies through registration. BMJ. 2024;384: e076123. 38195116 10.1136/bmj-2023-076123

[25] Wang SV, Pottegård A, Crown W, Arlett P, Ashcroft DM, Benchimol EI, et al. HARmonized Protocol Template to Enhance Reproducibility of hypothesis evaluating real-world evidence studies on treatment effects: A good practices report of a joint ISPE/ISPOR task force. Pharmacoepidemiol Drug Saf. 2023;32(1):44–55. 36215113 10.1002/pds.5507 PMC9771861

