# Supplementary material for: Analysis of indications for selectively missing results in comparative registry-based studies in medicine: a meta-research study
Source: Res Integr Peer Rev. 2025 Mar 5;10:2. doi: 10.1186/s41073-025-00159-x (PMC11881244; doi:10.1186/s41073-025-00159-x)
Supplement: Supplementary file 1 — Additional file 1. Search strings for selecting registry-based cohort studies comparing interventions. [file 41073_2025_159_MOESM1_ESM.docx]

Additional file 1: Search filter for non-randomized comparative study designs

| #1 | ((Registries[mesh] OR registry[tiab] OR registries[tiab] OR register[tiab] OR database[tiab]) NOT (("Trials Registry"[tiab] OR "Trial Registry"[tiab] OR "registry number"[tiab] OR "registry:"[tiab] OR registry;[tiab] OR "Trial Register"[tiab] OR "register number"[tiab] OR "register:"[tiab] OR register;[tiab] OR "number:"[tiab] OR number;[tiab] OR "database:"[tiab] OR database;[tiab] OR "Trials database"[tiab] OR "Trial database"[tiab) NOT (registry[ti] OR register[ti] OR "registry based"[tiab] OR "registry trial"[tiab] OR "national registry"[tiab] OR "national register"[tiab] OR "national registries"[tiab] OR "national database"[tiab] OR "patient registry"[tiab] OR "patient register"[tiab] OR "diseases registry"[tiab] OR "outcomes registry"[tiab] OR "clinical registry"[tiab] OR "clinical register"[tiab] OR "clinical data registry"[tiab]))) |
| --- | --- |
| #2 | (cohort[all] OR (control[all] AND study[all]) OR (control[tw] AND group*[tw]) OR epidemiologic studies[mh] OR program[tw] OR clinical trial[pt] OR comparative stud*[all] OR evaluation studies[all] OR statistics as topic[mh] OR survey*[tw] OR follow-up*[all] OR time factors[all] OR ci[tw]) NOT ((animals[mh:noexp] NOT humans[mh:noexp]) OR comment[pt] OR editorial[pt] OR review[pt] OR meta analysis[pt] OR case report[tw] OR consensus[mh] OR guideline[pt] OR history[sh]) |
| #3 | Therapeutics[mesh] OR “surgical procedures, operative"[mesh] |
| #4 | #1 AND #2 AND #3 |
