# Supplementary figures and images for: Analysis of indications for selectively missing results in comparative registry-based studies in medicine: a meta-research study
Source: Res Integr Peer Rev. 2025 Mar 5;10:2. doi: 10.1186/s41073-025-00159-x (PMC11881244; doi:10.1186/s41073-025-00159-x)

**Additional file 3**

**
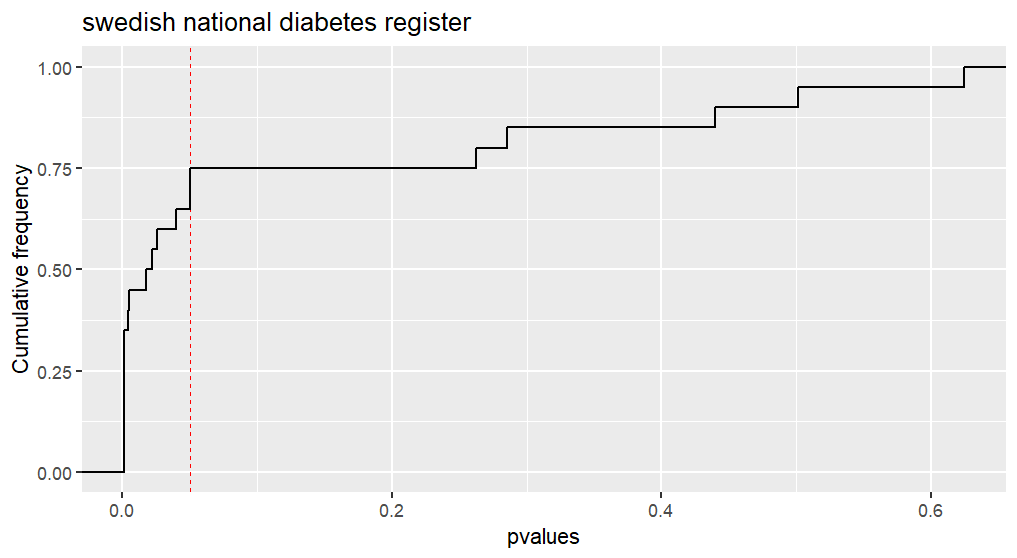
**

**
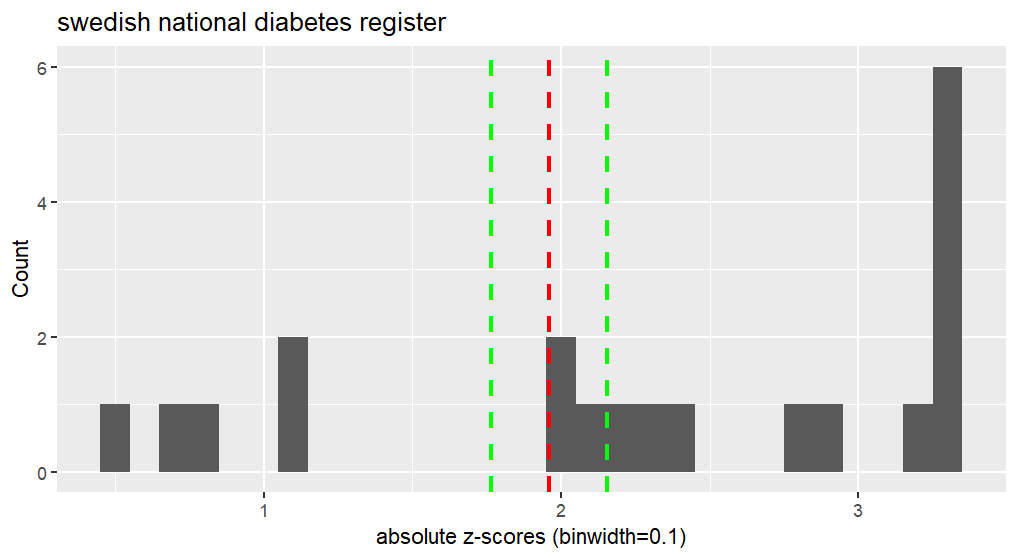
**

**
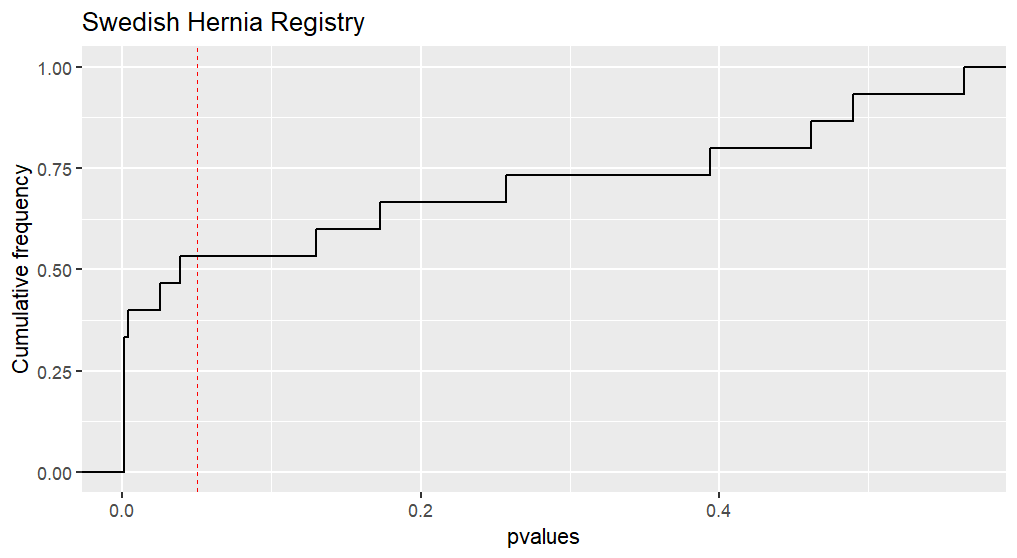

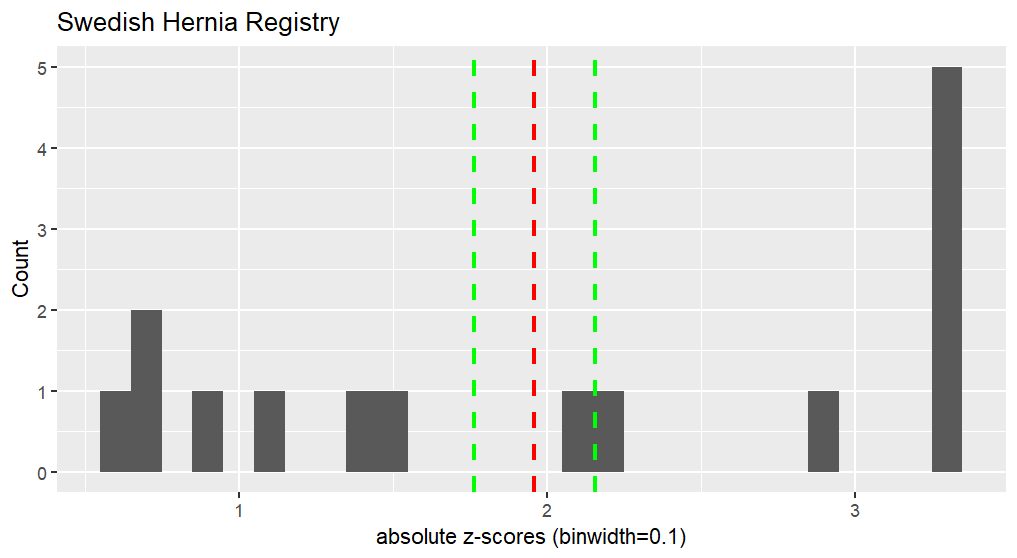
**

**
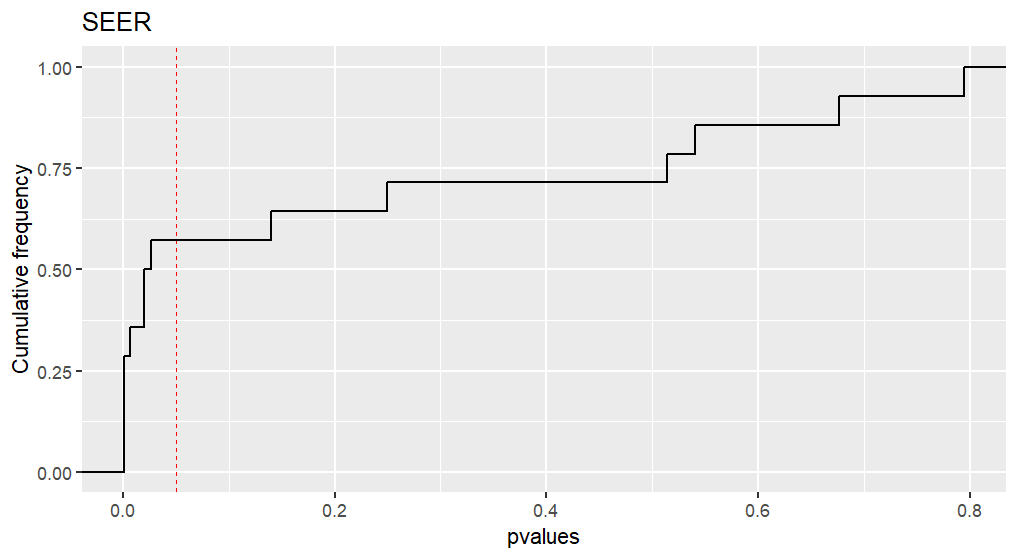
**

**
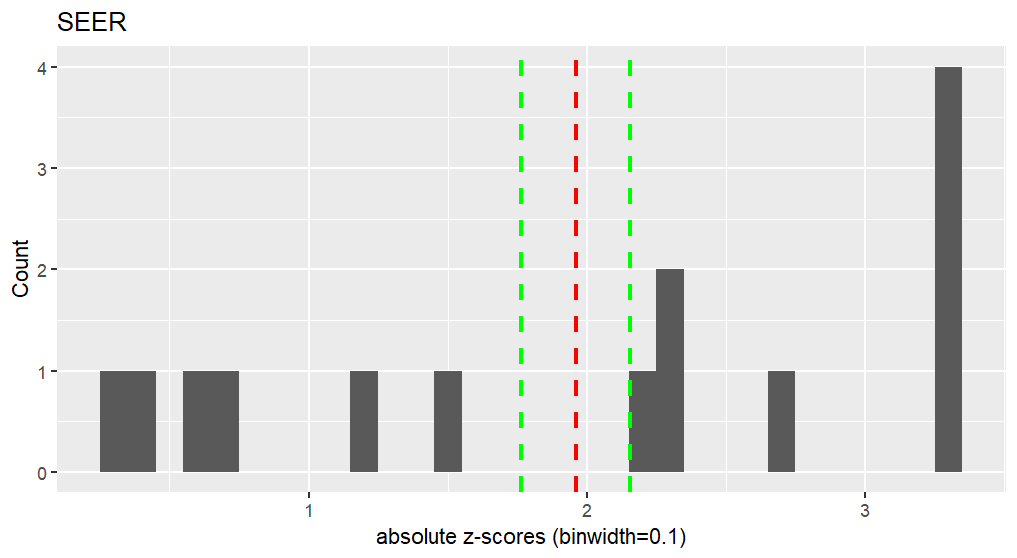
**

**
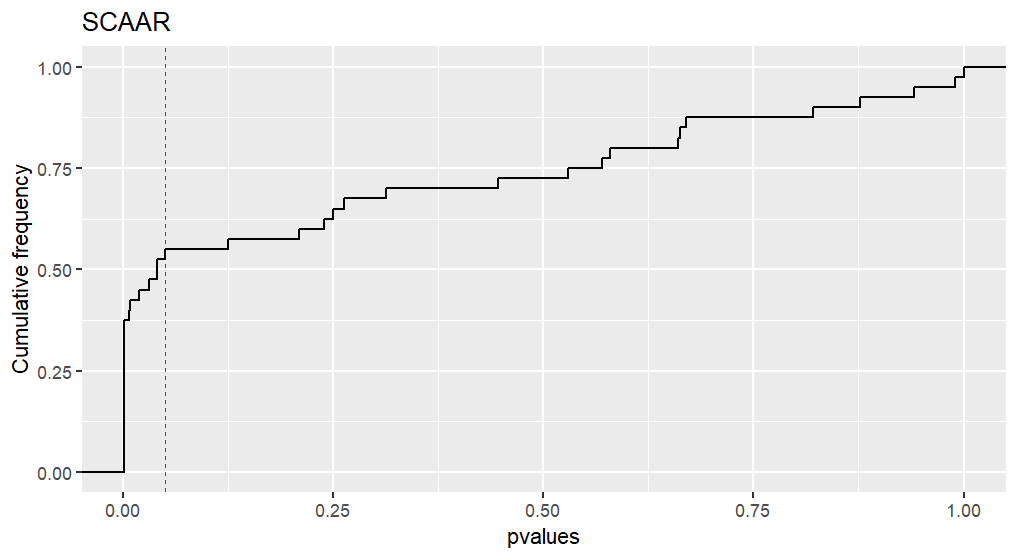
**

**
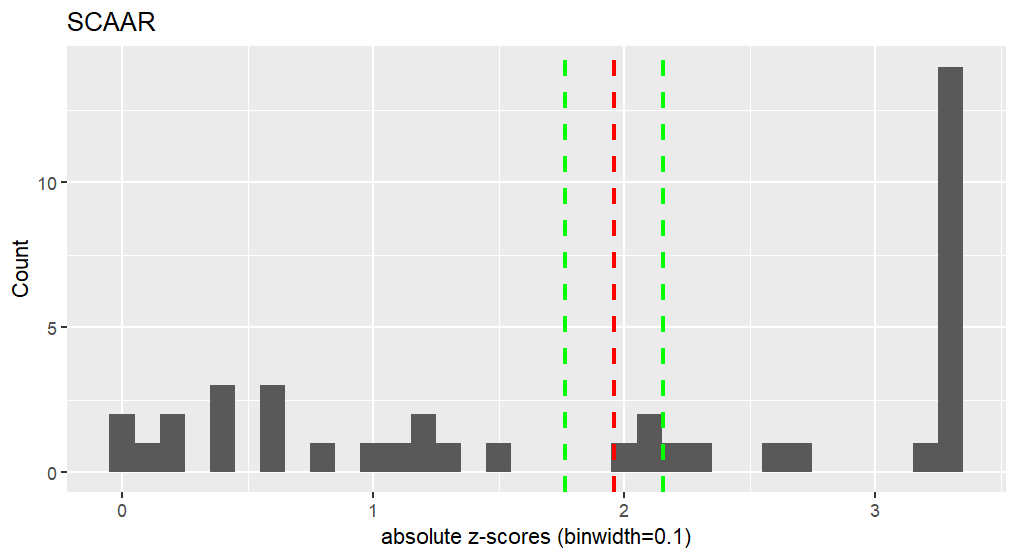
**

**
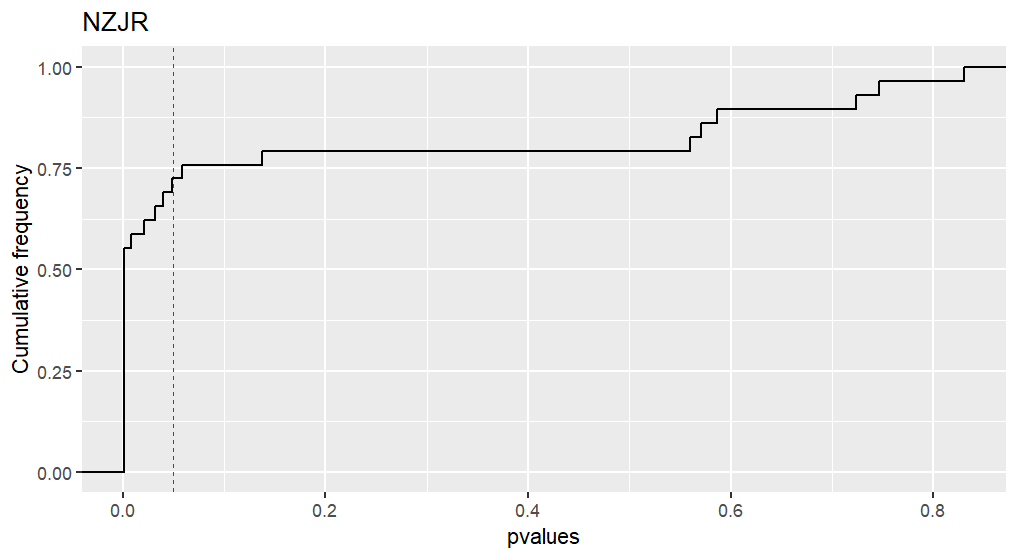
**

**
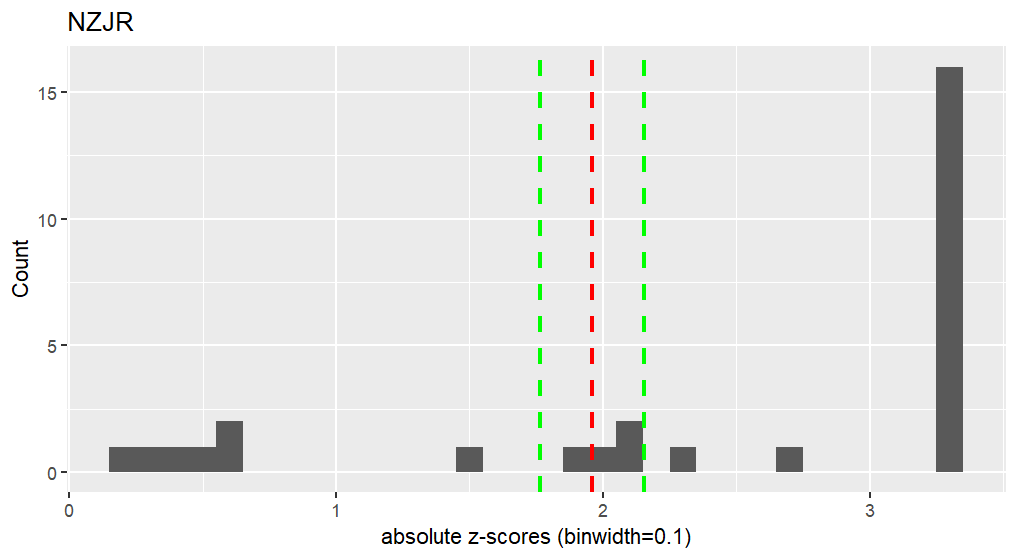
**

**
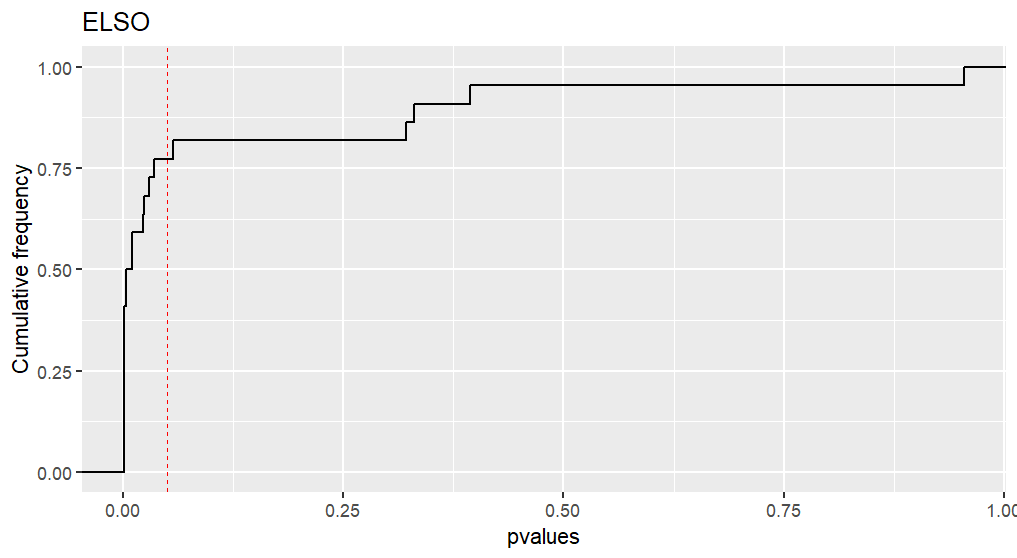
**

**
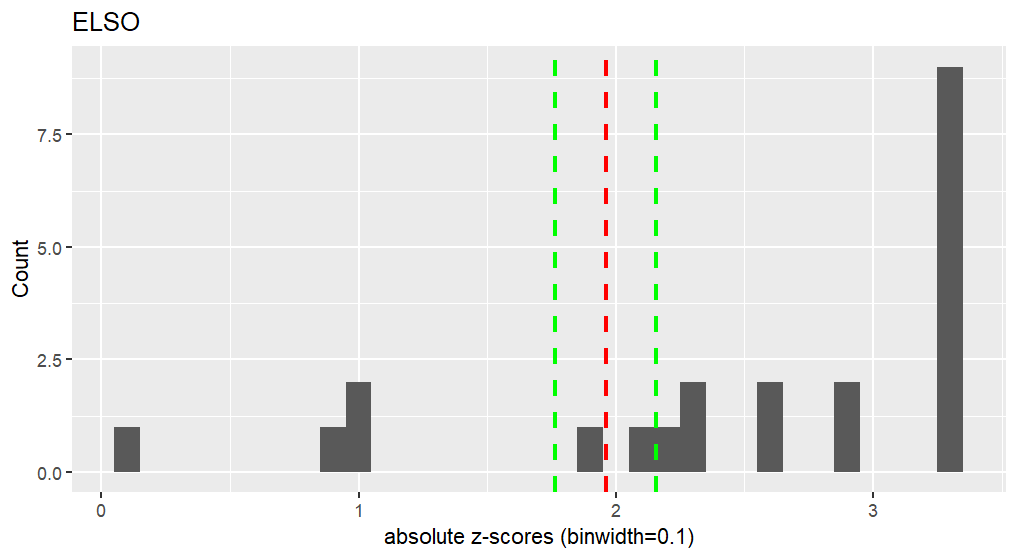
**

**
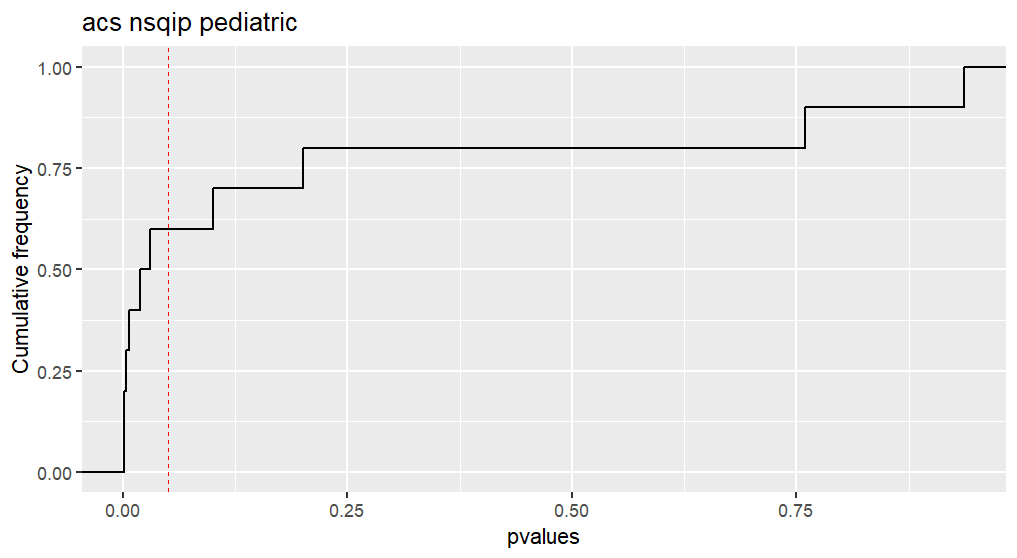
**

**
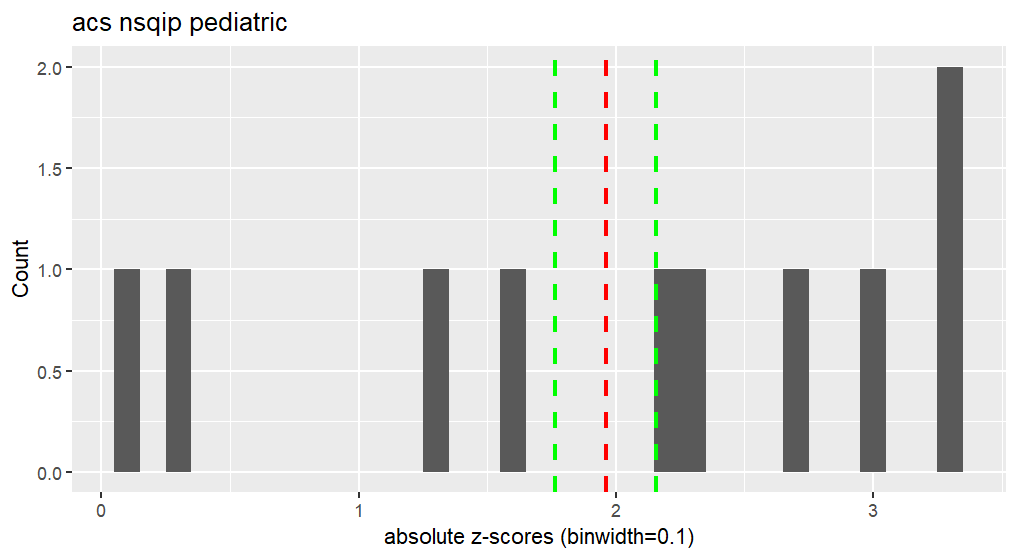
**

Supplement: Supplementary file 3 — Additional file 3. Sub-Plots for single registries. [file 41073_2025_159_MOESM3_ESM.docx]
